# Modulation of alternative splicing induced by paclitaxel in human lung cancer

**DOI:** 10.1038/s41419-018-0539-4

**Published:** 2018-04-30

**Authors:** Ziran Zhu, Dan Chen, Wenjing Zhang, Jinyao Zhao, Lili Zhi, Fang Huang, Haoyu Ji, Jinrui Zhang, Han Liu, Lijuan Zou, Yang Wang

**Affiliations:** 1 Institute of Cancer Stem Cell & Radiotherapy Oncology Department of the Second Affiliated Hospital, Dalian Medical University, Dalian, 116044 China; 2 Department of Pathology, the First Affiliated Hospital, Dalian Medical University, Dalian, 116011 China

## Abstract

Paclitaxel is utilized as the first-line chemotherapeutic regimen for the majority of advanced non-small-cell lung carcinoma. However, whether paclitaxel could suppress cancer progression through modulating RNA alternative splicing remains largely unknown. Here, we demonstrated the effects of paclitaxel on cell proliferation inhibition, cell cycle arrest, and apoptosis. Mechanistically, paclitaxel leads to transcriptional alteration of networks involved in DNA replication and repair, chromosome segregation, chromatin silencing at rDNA, and mitosis at the transcriptional level. Moreover, paclitaxel regulates a number of cancer-associated RNA alternative splicing events, including genes involved in cellular response to DNA damage stimulus, preassembly of GPI anchor in ER membrane, transcription, and DNA repair. In particular, paclitaxel modulates the splicing of ECT2, a key factor involved in the regulation of cytokinesis. Briefly, paclitaxel favors the production of ECT2-S, the short splicing isoforms of ECT2, thereby inhibiting cancer cell proliferation. Our study provides mechanistic insights of paclitaxel on RNA alternative splicing regulation, thus to offer a potential novel route for paclitaxel to inhibit cancer progression.

## Introduction

Lung cancer is one of the most common malignant cancers world-wide. In 2017, 222,500 new cancer cases and 155,870 deaths were estimated in lung and bronchus according to the American Cancer Society, in which, non-small-cell lung carcinoma (NSCLC) accounts for 83% of lung cancer^1^. Most patients with stage III and IV NSCLC receive chemotherapy with or without radiation. Paclitaxel, a microtubule inhibitor, is commonly used in advanced NSCLC treatment either in combination with platinum-based agents or as monotherapy^2^. It is well established that Paclitaxel functions by directly binding to polymerized β-tubulin, thereby resulting in perturbation of microtubules dynamics^3^. Paclitaxel inhibits the dynamic instability of the mitotic spindle, leading to impaired chromosome alignment^4^. Consequently, cells are arrested by the spindle checkpoint at the G2/M phase and eventually go through apoptosis^5^. Multiple cellular pathways participate in paclitaxel-induced cytotoxicity, including RAS, MYC-controlled pathway, and inhibition of spleen tyrosine kinase^6–8^. However, further molecular mechanisms about paclitaxel might need to be discovered due to the clinical complexity.

Almost 95% of genes are alternatively spliced in humans. Importantly, alternative splicing (AS) of pre-messenger RNA (mRNA) leads to the production of multiple mature mRNAs and protein isoforms with distinct structural and functional properties^9^. Dysregulation of AS also leads to aberrant protein isoforms, which may contribute to tumor initiation, progression, and therapeutic treatments difficulties^10, 11^. Previously, in NSCLC, some pre-mRNA splicing regulators have been demonstrated to be abnormally expressed, including SRSF1, SRSF2, SRPK1, and SRPK2^12^. In addition, Shultz et al.^13^ employed RNA oligonucleotides to modulate caspase 9 pre-mRNA splicing in favor of caspase 9b production, resulting in an increase in the IC50 of non-small-cell lung cancer cells to paclitaxel. Moreover, in paclitaxel resistant triple-negative breast cancer cells, aberrant RNA splicing was defined, and the interaction of TRA2A with the splicing factor hnRNP M can co-regulate AS^14^. Taken together, these data suggest that as a chemotherapeutic agent, paclitaxel may suppress cancer cell proliferation by modulating the AS of cancer-related genes. However, the mechanistic details underlying such splicing regulation upon paclitaxel treatment are still largely unknown.

Here, we report that paclitaxel can function as a chemotherapeutic drug by modulating cancer-related splicing events in lung cancer cells. To systematically identify the splicing events regulated by paclitaxel, we performed RNA-Seq on paclitaxel treated or control lung cancer cells. Interestingly, in addition to gene expression changes, we discovered that extensive AS occurred upon paclitaxel treatment. Briefly, we identified 994 significantly differentially expressed genes, and 855 AS events after paclitaxel treatment in A549 cells. Our results suggested that paclitaxel predominantly induced skipped exons compared to other splicing types. We identified distinct AS events of FMNL3, ZMIZ2, ECT2, PLD2, and DDIT3, which may be involved in cancer cell proliferation, epithelia-mesenchymal transition and actin cytoskeleton organization. Most importantly, lung cancer cells expressing ECT2-S appeared to be more sensitive to paclitaxel. Taken together, our results not only identified mRNAs regulated by paclitaxel in NSCLC, but also suggest that AS switch might provide an alternative new route for paclitaxel to suppress cancer progression.

## Results

### Paclitaxel inhibits cell proliferation and induces cell cycle arrest and apoptosis

**Fig. 1:**
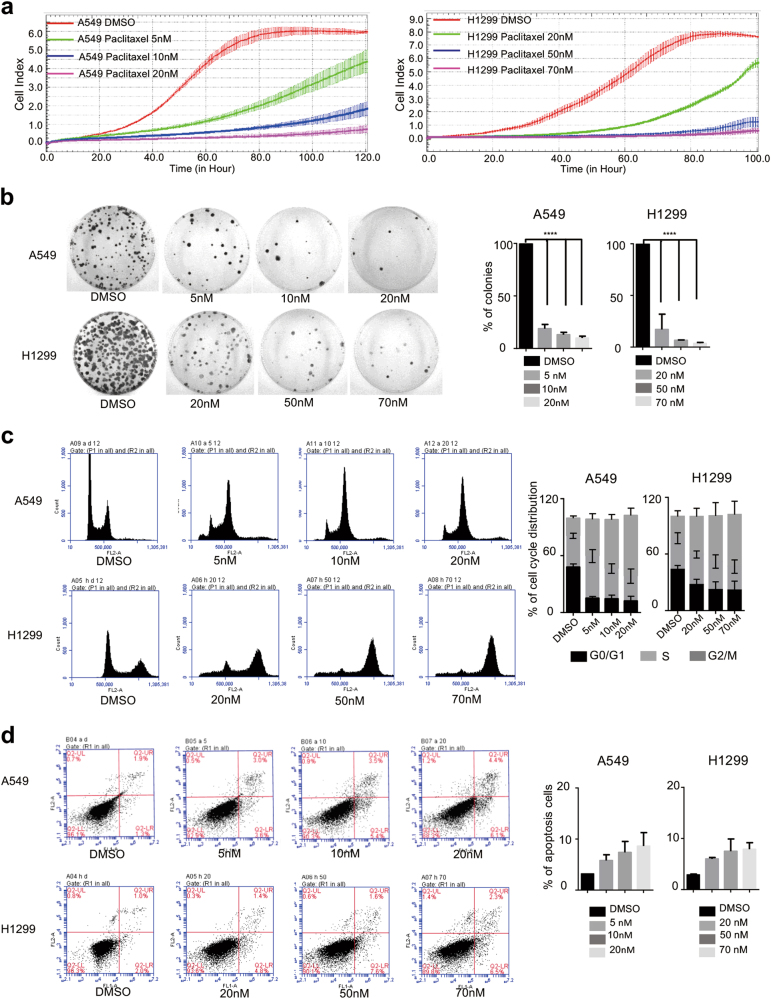
Paclitaxel inhibits cell proliferation and induces cell cycle arrest and apoptosis. **a** Growth curve assays were conducted to evaluate the effects of paclitaxel on the proliferation of the non-small-cell lung carcinoma cell lines A549 and H1299. **b** Colony formation assays were performed in A549 and H1299 cells treated for 24 h with different concentrations of paclitaxel. Representative pictures of the whole plates are shown. Three experiments were carried out with corresponding data normalized to control and expressed as mean +/- SD (***indicated *p* < 0.001). **c** Flow cytometry approach was utilized to analyze the cell cycle and apoptosis **d** of cells treated with different dosages of paclitaxel (5, 10, 20 nM for A549 cells) and (20, 50, 70 nM for H1299 cells) for 12 h respectively. *indicated *p* < 0.05

We sought to investigate the effect of paclitaxel on lung cancer cells. To this end, cell viability was determined by MTT assay after 48 h treatment with different concentrations of paclitaxel and the IC50 of A549 and H1299 cells were 7.22 nM and 40.78 nM, respectively (Supplementary Fig. 1a). Importantly, paclitaxel exhibits potent cell cytotoxicity in a dose-dependent manner, as demonstrated in two lung cancer cell lines, including A549 and H1299. Briefly, A549 cells were incubated with paclitaxel for 24 h at different concentrations (5, 10, 20 nM), whereas H1299 cells were treated with paclitaxel at 20, 50, 70 nM for 24 h. Subsequently, cells were collected to analyze the cell proliferation and growth as judged by real-time cell analyzer (RTCA) and colony formation assay (Fig. 1a, b). The treatment with 5 nM of paclitaxel showed an obvious inhibition of cell growth and higher concentrations (10 and 20 nM) showed much stronger inhibition. Similar phenotypes were also obtained from H1299 cells (Fig. 1a, b). In addition, cell migration was suppressed after paclitaxel treatment (Supplementary Fig. 1b). Moreover, when treated with distinct concentrations of paclitaxel, A549 and H1299 cells demonstrated a dose-dependent cell cycle arrest at G2/M phase, as well as a decreased G1 phase, as judged by FACS (Fig. 1c). We also measured the apoptosis, and found that about 10% apoptotic population was achieved at the maximum concentration of paclitaxel treatment in A549 and H1299 cells, respectively (Fig. 1d). Taken together, we demonstrated that paclitaxel potently induced cell growth inhibition, G2/M cell cycle arrest, and apoptosis in lung cancer cells.

### Systematic identification of gene expression changes induced by paclitaxel

**Fig. 2:**
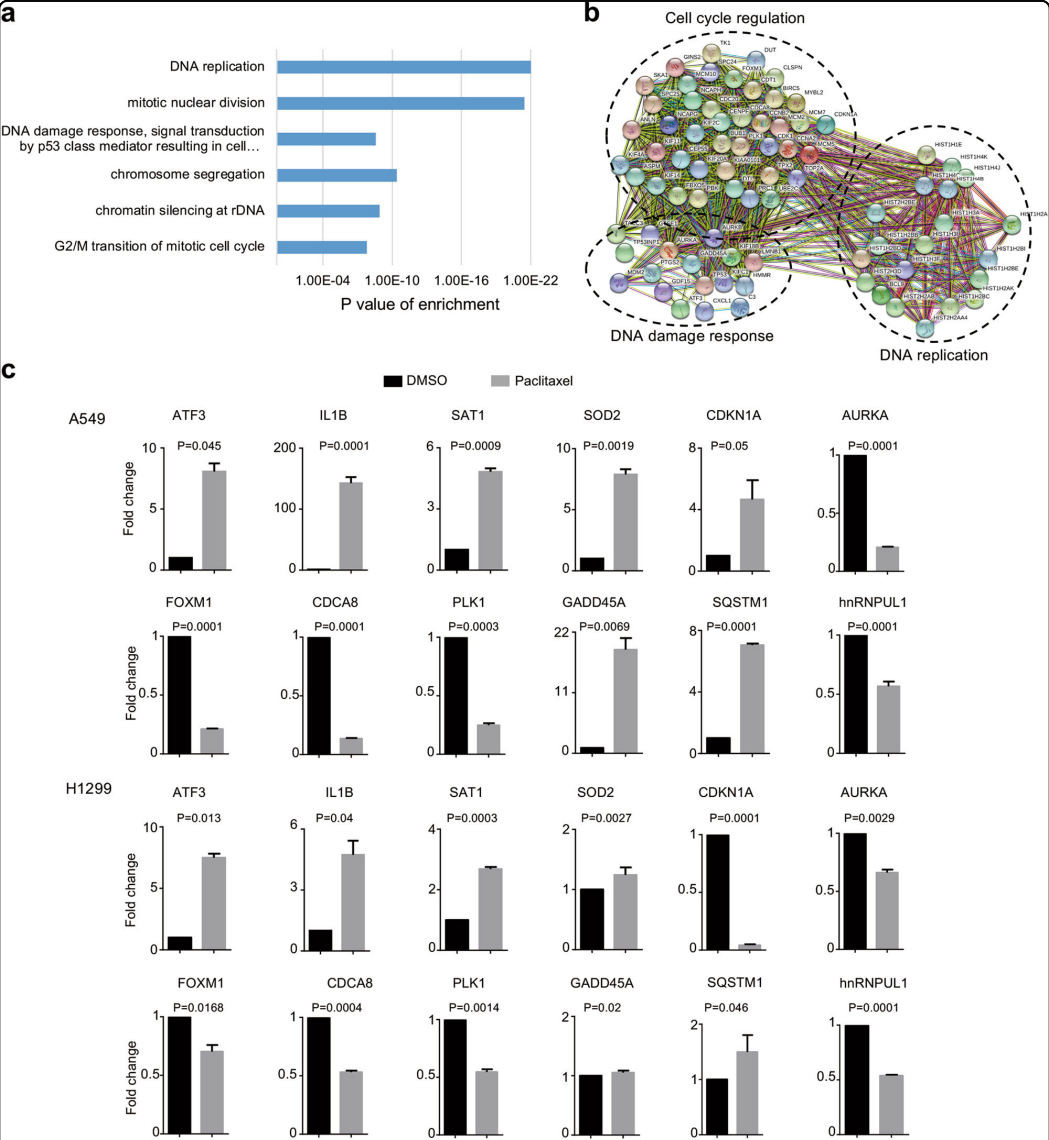
Systematic identification of gene expression changes induced by paclitaxel. **a** Gene ontology analyses of paclitaxel induced gene expression events. Fisher exact *p*-values were plotted for each category. **b** The functional association networks of paclitaxel-induced genes were analyzed using the STRING database, with subgroups marked by their functions. **c** Validation of gene expression changes by real-time RT-PCR. The mean and SD of relative fold changes from triplicate experiments were plotted, with *p*-values calculated by paired Student’s test

To further investigate the molecular mechanisms of paclitaxel-induced cell growth inhibition, we performed high-throughput mRNA sequencing (mRNA-seq) with A549 cells treated by paclitaxel at 7.22 nM for 72 h. Strikingly, we identified 994 genes with significant expression change (twofold with adjusted *p* < 0.05). Subsequent gene ontology analysis showed that DNA replication, mitotic nuclear division, DNA damage, chromosome segregation, and G2/M transition of mitotic cell cycle were the most enriched pathways (Fig. 2a). In addition, most of paclitaxel-induced genes were functionally associated with networks that contains genes involved in cell cycle regulation, DNA damage response and DNA replication, as judged by the Search Tool for the Retrieval of Interacting Genes/Proteins (STRING) (Fig. 2b). We further randomly validated several targets induced by paclitaxel with real-time reverse transcription (RT)-PCR (Fig. 2c). Among these genes, GADD45A, AURKA are involved in DNA damage and regulation of apoptotic process. CDKN1A, AURKA, FOXM1, and PLK1 are involved in the regulation of cell cycle. Interestingly, some of the splicing factors were also affected by paclitaxel, such as hnRNPUL1 for example, indicating that paclitaxel might also inhibit cancer cell growth through modulating RNA AS.

### Global regulation of AS induced by paclitaxel in cancer-related genes

**Fig. 3:**
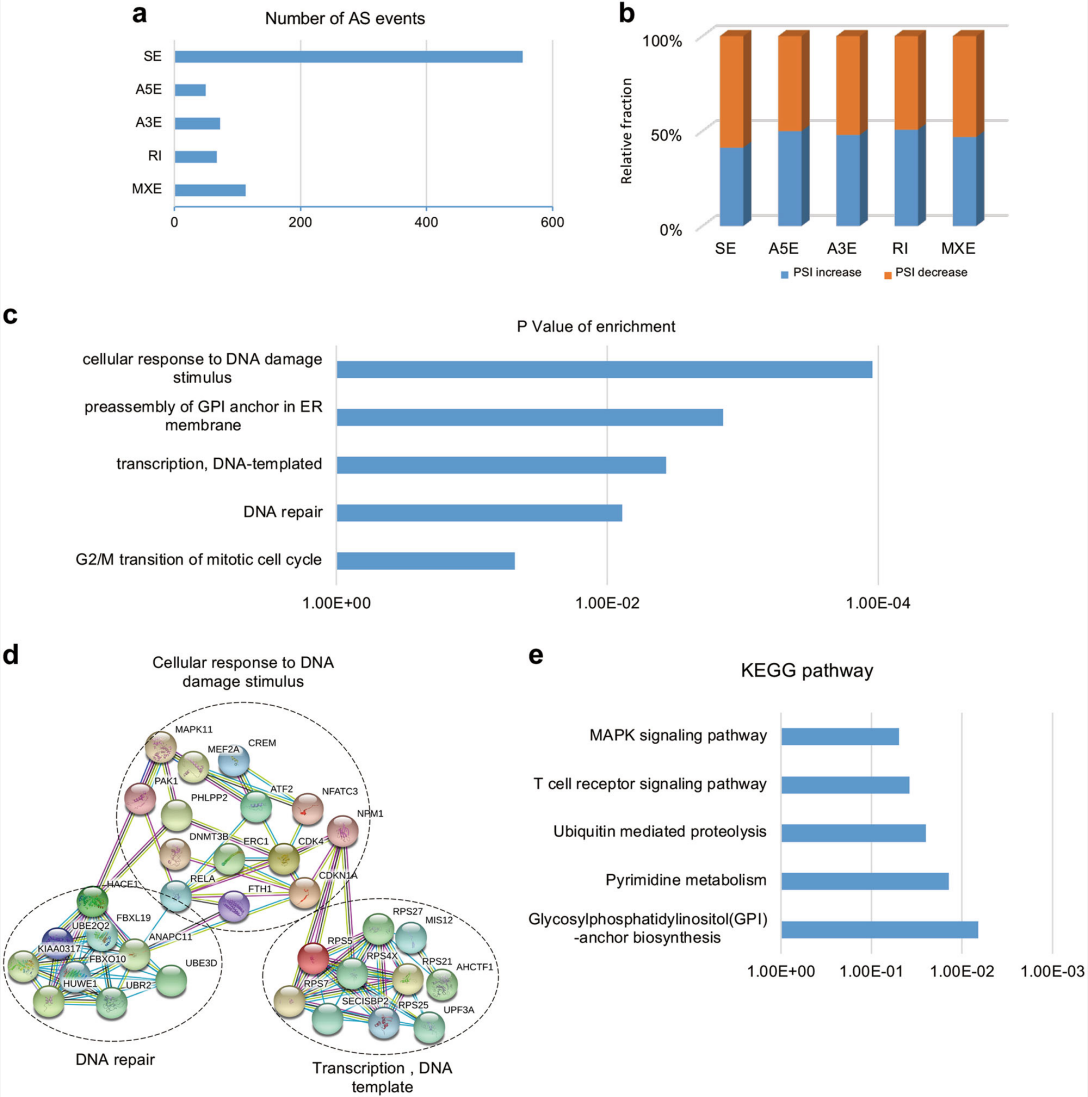
Global splicing regulation induced by paclitaxel . **a** Quantification of different AS events induced by paclitaxel. **b** The relative fraction of each category of AS event affected positively or negatively by paclitaxel. **c** Gene ontology analysis of paclitaxel-induced AS targets. Fisher exact *p*-values were plotted for each category. **d** Functional association networks of paclitaxel-induced AS targets were analyzed using the STRING database, and subgroups are marked according to their functions. **e** Pathways were analyzed by KEGG pathway database for paclitaxel-induced AS targets

To explore the effects of paclitaxel on alteration of AS, we systematically analyzed the mRNA-seq data to identify differentially changed AS events. As expected, we obtained 855 paclitaxel-regulated AS events with an obvious change of percent-spliced-in (PSI) values (the change of PSI > 0.15), including 552 skipped exons (SE), 50 alternative 5′ ss exon (A5E), 73 alternative 3′ ss (A3E), 67 retained intron (RI), and 113 mutually exclusive exons (MXE) (Fig. 3a). Subsequent analysis indicated that the majority of AS events were negatively regulated in cells treated with paclitaxel (Fig. 3b).

Using gene ontology (GO) analysis, the paclitaxel-induced splicing events were enriched in groups related to cell response to DNA damage stimulus, preassembly of GPI anchor in ER membrane, DNA-templated transcription, DNA repair and G2/M transition of mitotic cell cycle (Fig. 3c). These targets are consistent with the diversity of global gene expression induced by paclitaxel. In addition, we conducted STRING analysis and found that many of the paclitaxel-induced splicing targets were functionally connected into well-linked interaction networks, including genes involved in DNA damage, DNA repair, and DNA template-dependent transcription (Fig. 3d). Meanwhile, we performed KEGG analysis and found that interactions induced by paclitaxel were focused on MAPK signaling pathway, T-cell receptor signaling pathway and GPI-anchor biosynthesis (Fig. 3e). Collectively, these results suggested that the biological processes affected by paclitaxel are related to DNA damage, DNA repair, and cell cycle, which is consistent with functions of paclitaxel on microtubules.

### Alternative splicing switch induced by paclitaxel

**Fig. 4:**
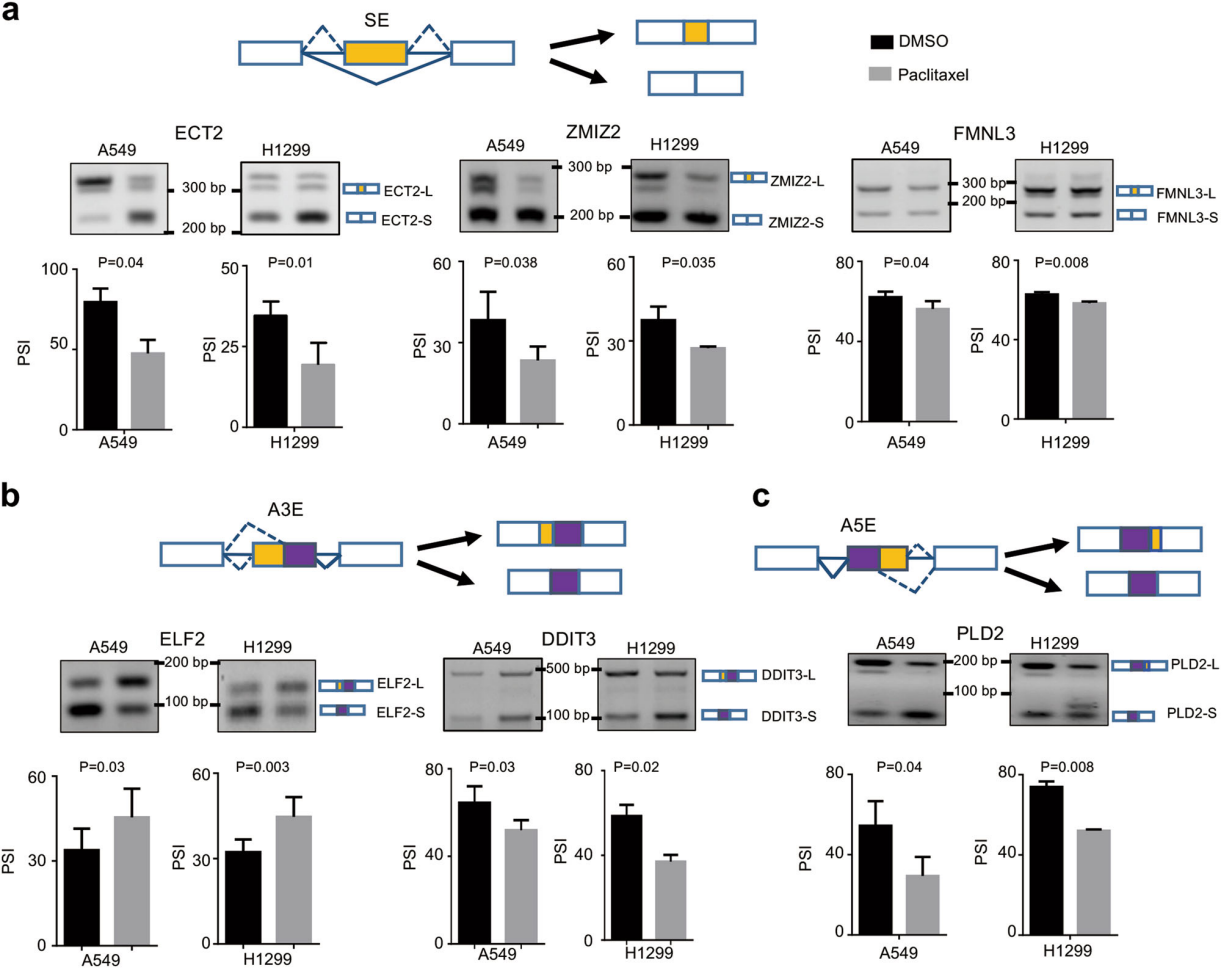
Alternative splicing switch induced by paclitaxel. **a** Exons skipping in ECT2, ZMIZ2, and FMNL3 were examined in A549 and H1299 cells upon treated with paclitaxel. **b** Alternative 3′ splice sites usage of ELF2 and DDIT3 were determined in A549 and H1299 cells when treated with paclitaxel. **c** Alternative 5′ splice sites usage of PLD2 were investigated in A549 and H1299 cells when treated with paclitaxel. Representative agarose gel figures of RT-PCR validations in A549 and H1299 cells were shown. The mean+/-SD of PSIs from three experiments were plotted, *p*-values were calculated by paired Student’s test

We subsequently validated the splicing switch of six randomly chosen targets from the mRNA-seq results. As expected, we demonstrated that exon 4 skipping of ECT2 (GEF epithelial cell transforming sequence 2) was significantly induced by paclitaxel. Similarly, the skipping of exon 8 of ZMIZ2 (zinc finger MIZ-type containing 2) and exon 6 of FMNL3 (formin-like 3) were also elevated after paclitaxel treatment (Fig. 4a and Supplementary Fig. 2). Meanwhile, we further verified that paclitaxel promoted the upstream 3′ ss usage of ELF2 (ETS transcription factor 2) and DDIT3 (DNA damage inducible transcript 3) (Fig. 4b and Supplementary Fig. 2), whereas paclitaxel inhibited the distal 5′ ss usage of PLD2 (phospholipase D2) (Fig. 4c). Among these genes, ECT2 regulates cytokinesis in non-transformed cells by acting RhoA^15, 16^. ECT2 is overexpressed in multiple cancers^17–19^. It is reported that ECT2 depletion impairs tumorigenic growth of NSCLC cell^20^. ZMIZ2 plays a role in regulating the activity of the Wnt/β-catenin signaling pathway^21^. FMNL3 is involved in regulation of cell morphology and cytoskeletal organization^22^. Thus, we next sought to investigate whether the splicing switch of ECT2 is indeed involved in the paclitaxel-mediated cancer inhibition.

### ECT2 splicing switch participated in paclitaxel-induced lung cancer inhibition

**Fig. 5:**
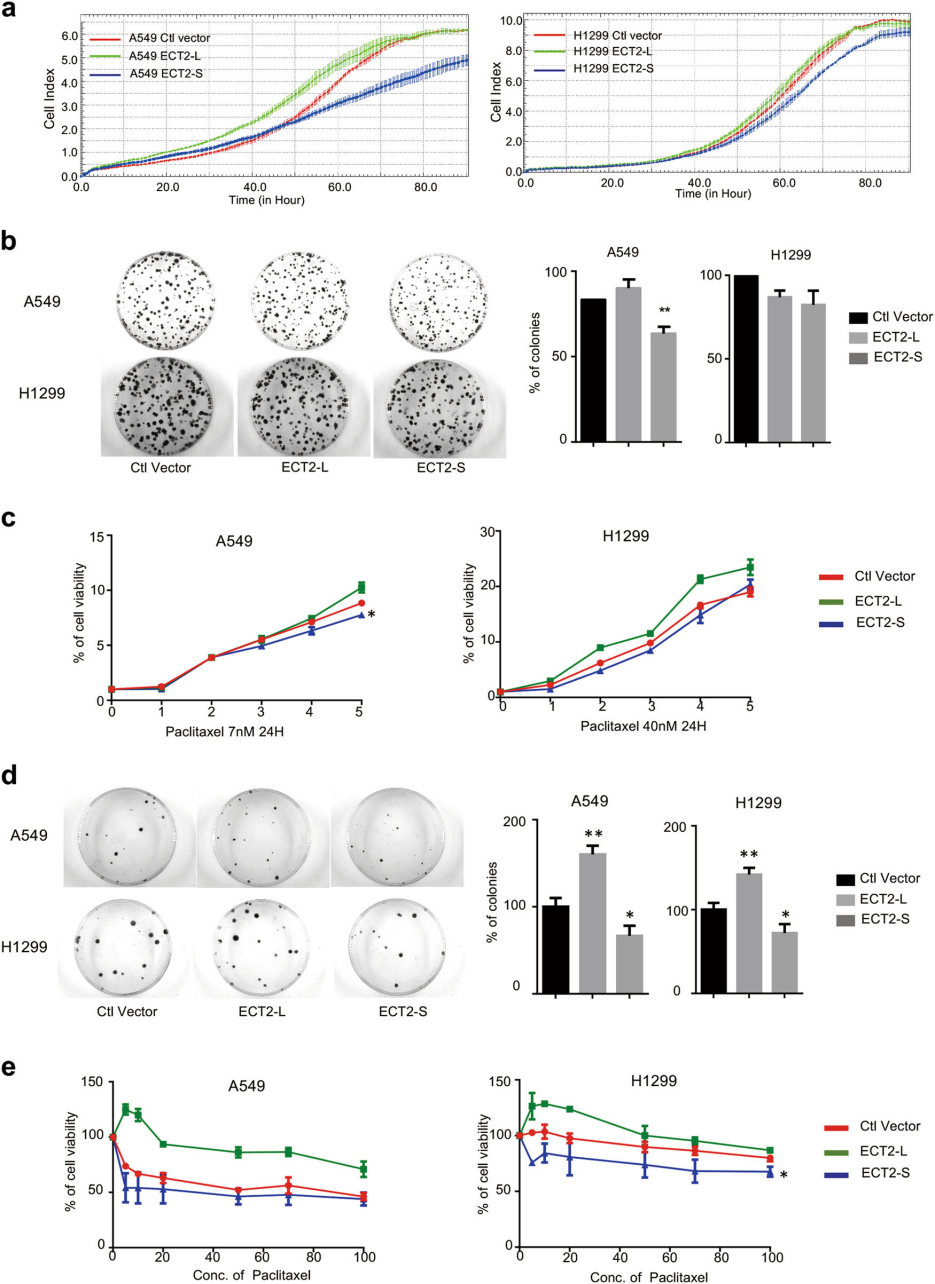
ECT2 splicing switch participated in paclitaxel-induced lung cancer inhibition. **a** Cell proliferation of A549 and H1299 cells stably expressing ECT2-L or ECT2-S were analyzed using RTCA assay. **b** The growth of A549 and H1299 cells stably expressing ECT2-L or ECT2-S were analyzed by colony formation assay. **c** A549 and H1299 cells stably expressing ECT2-L or ECT2-S were incubated with 7.22 nM or 40 nM paclitaxel for 24 h, respectively, and then the cell viability and growth were analyzed by CCK8 and colony formation assays (**d**). The mean+/-SD of relative colony numbers were plotted, with *p*-values calculated by Student’s test. **e** A549 and H1299 cells stably expressing ECT2-L or ECT2-S were treated with different concentrations of paclitaxel, and the cell viability was examined by CCK8 assay

We have demonstrated that paclitaxel induced the skipping of exon 4 of ECT2, which resulted in a frame shift in the BRCT domain of ECT2 (Fig. 4a). Importantly, ECT2 was reported to promote nucleotide exchange on Rho family members of small GTPase, known to regulate ribosomal RNA (rRNA) synthesis through a PKC-Ect-Rac1-NPM signaling axis that is required for lung tumorigenesis. Therefore, we aimed to determine the role of the short isoform of ECT2-S in cancer progression, especially in paclitaxel-mediated cancer suppression. To this end, we stably expressed two isoforms of ECT2, ECT2-L, or ECT2-S, in A549 and H1299 cells (Supplementary Fig. 3a, b). We treated cells with paclitaxel and found that ECT2-L was predominantly nucleus-localized, whereas ECT2-S was distributed in both cytoplasm and nucleus as examined by immunofluorescence (Supplementary Fig. 3c). Strikingly, we found cells expressing ECT2-S grew evidently slower than control and ECT2-L expressing cells as judged by RTCA and colony formation assays (Fig. 5a, b). These data suggested that ECT2-S, the short isoform, could inhibit cancer cell proliferation, which has the opposite function to the canonical full-length ECT2.

Subsequently, we investigated whether ECT2-S can suppress cancer cell growth synergistically with paclitaxel. To this end, we treated cells stably expressing ECT2-L or ECT2-S with paclitaxel for 24 h respectively. We further applied the resulting cells to examine the cell viability by using the CCK8 and colony formation assays. As expected, A549 cells stably expressing ECT2-S could significantly inhibit cancer cell growth as compared to control cells, whereas cells expressing ECT2-L could promote cancer cell growth compared to control cells (Fig. 5c, d). Consistently, such results were also confirmed in another lung cancer cell line, H1299 (Fig. 5c, d). To further validate the synergistic effect of ECT2-S with paclitaxel, we treated cells stably expressing ECT2-S or ECT2-L with different concentrations of paclitaxel and examined the cell viability. The data demonstrated that the combined treatment of ECT2-S with paclitaxel could markedly inhibit cancer cell proliferation in both A549 and H1299 cells (Fig. 5e). Collectively, ECT2 splicing switch participated in paclitaxel-induced lung cancer inhibition.

### ECT2-S is reduced in cancer samples and negatively correlated to survival

**Fig. 6:**
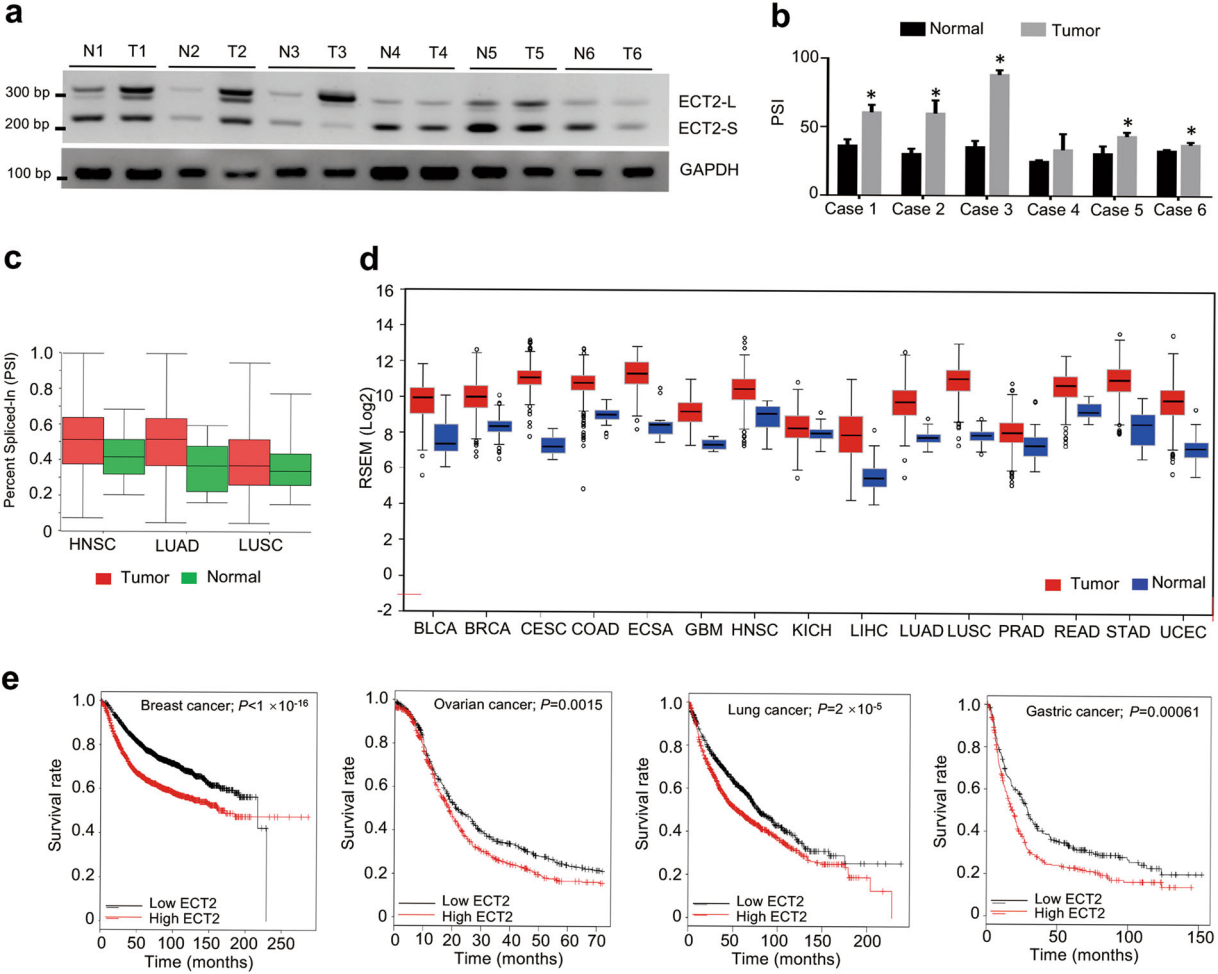
ECT2-S is reduced in cancer samples and ECT2 is negatively correlated to survival. **a** Total RNAs isolated from six paired NSCLC tumors and adjacent normal tissues were examined to measure the splicing of ECT2 by semi-quantitative RT-PCR. The mean+/- SD from three experiments was plotted. * indicated *p* < 0.05 (**b**). **c** The splicing change of ECT2 was assayed in multiple cancers by analyzing the TCGA consortium containing RNA-seq datasets from thousands of patients. **d** The expression levels of ECT2-L were analyzed in various large scale studies obtained from TCGA dataset. **e** Kaplan–Meier curve showing overall survival of patients with lung, breast, ovarian, and gastric cancers bearing high or low ECT2-L expression

To further determine the clinical significance of ECT2 splicing in patients, we tested the relative levels of two ECT2 isoforms in paired lung cancer samples and the adjacent normal tissues surgically obtained from six patients. As compared to the paired normal samples, the relative mRNA levels of ECT2-S were significantly reduced in five out of six tested primary NSCLC specimens (Fig. 6a, b), suggesting a common reduction of ECT2-S expression despite obvious heterogeneity in different tumor samples. Consistent with our results, analyses of RNA-seq datasets from the TCGA consortium demonstrated that ECT2-S levels were markedly decreased in lung adenocarcinoma, lung squamous carcinoma, and head and neck cancer (Fig. 6c).

Additionally, we also analyzed the expression level of ECT2-L in various large scale studies from TCGA, and found ECT2-L is evidently elevated in multiple cancers, including lung, breast, colon, renal, esophageal, liver cancer, and so on (Fig. 6d and Supplementary Fig. 4a–d).

Intriguingly, we applied Kaplan–Meier Plotter to analyze the overall survival of cancer patients with distinct ECT2-L levels using datasets from large scale screening. As expected, higher expression of ECT2-L was related to poor overall survival in patients with lung, breast, ovarian, and gastric cancer (Fig. 6e and Supplementary Fig. 4e). These data further proved that paclitaxel might suppress cancer progression through mediating ECT2 splicing.

## Discussion

Paclitaxel remains the first-line standard therapy regimen with platinum agent or singly in the second-line setting for advanced and metastatic NSCLC. Paclitaxel suppresses tumorigenesis primarily through interfering with mitotic spindle dynamics and triggering the mitotic checkpoint, thereby inducing an extended G2/M arrest. Thus, such cycle arrest can lead to cell death via the mitochondrial apoptotic pathway. Aberrant AS exerts control over major hallmarks of cancer, including apoptosis, epithelial-mesenchymal transition, and tumor proliferation and invasion. Moreover, numerous splicing variants of specific genes serve as molecular markers of cancer or directly mediate cancer pathogenesis^23–26^. Accumulating evidence suggests that different alternatively spliced transcripts were involved in response to clinical therapeutic drugs. In our model, we demonstrated that paclitaxel could inhibit cancer progression through regulating the AS of many cancer-related genes. Specifically, paclitaxel could switch the splicing of ECT2 from the canonical full-length isoform towards the short isoform ECT2-S, which in turn inhibit cancer cell proliferation. That is because normally the phosphorylation of ECT2 will activate Rho family GTPase to promote cancer progression. However, the skipping of exon 4 results in a disruption of the BRCT domain of ECT2, which offers a binding pocket for phosphorylated residues. Thus, the short isoform of ECT2 inhibits tumorigenesis specifically when treated with paclitaxel. Importantly, ECT2 has been shown to be involved in Wingless/Wnt signaling pathway, and controlling cleavage furrow formation during cytokinesis^27, 28^. Therefore, our results represent a novel mechanism for paclitaxel to suppress cancer progression through modulating AS of cancer-related genes.

In addition to ECT2 splicing, other splicing events might also contribute the suppression of tumorigenesis upon paclitaxel treatment. Using RNA-seq analysis, we also found that paclitaxel mediated some splicing events for several genes associated with DNA damage, transcription, DNA repair, and G2/M transition of mitotic cell cycle. For instance, paclitaxel controls splicing of FMNL3 and PLD2, which play critical roles in the regulation of cytoskeletal organization. These data suggest that paclitaxel regulates many splicing events critical to tumorigenesis, which might provide new therapeutic combinations to sensitize cancer cells to paclitaxel treatment.

## Materials and Methods

### Cell culture and treatment

Human lung cancer A549 and NCI-H1299 cell lines were obtained from the American Type Culture Collection (Manassas, VA, USA). Paclitaxel (S1150) was purchased from Sellect.cn. These cells were maintained in F12K Kaighn’s Modification and RPMI-1640 supplemented with 10% Fetal Bovine Serum (FBS) respectively. To stably overexpress ECT2-L/S in A549 and H1299 cells, we used lentiviral vectors. We transfected 293t cells with pCDH-3 × Flag-ECT2-L/S or pCDH-3 × Flag empty vectors according to the manufacture’s protocols. We collected the supernatant media containing virus by centrifugation to remove any cellular contaminant. Further, A549 and H1299 cells were infected with the viral particles, and the stably integrated cells were selected with 5 µg/ml puromycin for 5 days. Then cells were maintained in medium containing 2 µg/ml puromycin. All cells were maintained at 37 °C in a humidified incubator with 5% CO_2_. The expression of transgenes was confirmed by western bolts and RT-PCR before further analysis.

### Cell cycle and apoptosis analysis by flow cytometry

Cells were seeded in 60 mm-dish (160,000 cells/per dish) and allowed to grow overnight for 70% confluency. To analyze the cell cycle regulation induced by paclitaxel, A549 or H1299 cells were treated with paclitaxel, at the final concentration of 5, 10, 20 nM and 20, 50, 70 nM, respectively for 12 h. Cells were harvested and washed by PBS and then fixed with 75% cold ethanol overnight. Before analyzed by flow cytometry, we incubated the cells with RNase in 37 °C water for 30 min, stained with propidium iodide for 30 min on ice and in dark. Different cell populations were quantified based on DNA contents to discern cells with 2N (G1), S-phase, and 4N (G2/M), and fragment DNA (apoptotic cells). To illustrate apoptosis effects by paclitaxel, cells were treated for 24 h, then dissociated from culture plates using trypsin without EDTA. Cells were washed with PBS for two times and re-suspended in binding buffer and stained with Annexin V and propidium iodide prior to analysis using flow cytometry.

### RT-PCR and quantitative RT-PCR validation

We extracted total RNA from cells treated with or without paclitaxel using Trizol reagent (Invitrogen) according to the manufacturer’s instructions. Genome DNAs were removed by 30 min DNase I (Takara) treatment at 37 °C and then heat inactivation using 50 nM EDTA. Total RNA (2 µg) was then reverse-transcribed with PrimeScript RT reagent kit (Takara) with random primer, and 1 µl of the cDNA was used as the template for PCR amplification. RT-PCR products were separated on 3% gels. The amount of each splicing isoform was measured by comparison of the integrated optical density of detected bands using the GIS 1D Gel Image System (ver. 4.2; Tanon). The primers used for gene expression are shown in table. Real-time quantitative PCR was performed using Takara SYBR II kit on system according to the manufacturer’s instructions.

### Western blot

Cells were harvested with RIPA lysis buffer containing 1 mM Na_3_VO_4_, 1 mM Cocktail and 1 mM PMSF. Cell debris was removed by centrifugation. The protein samples (20 µg) were boiled of 5 min in 1 × SDS sample buffer and fractionated by 10% sodium dodecyl sulfate polyacrylamide gel electrophoresis and transferred to nitrocellulose membrane. The following antibodies were used: Flag (Sigma), TUBULIN (T5168). Bound antibodies were visualized with the ECL kit (ncmbio).

### RNA-sequencing analysis

A549 cells were treated with paclitaxel 7.22 nM or DMSO for 72 h, and then total RNAs were extracted using Trizol. We used the Illumina TruSeq Total RNA Sample Prep kits to purify poly-adenylated RNA (not exceeding 2 µg) after RNase free DNase digested as per manufactures instruction. Cytoplasmic rRNA was removed by the Ribo-Zero Human. Prior to generation of cDNA library with bar coded ends the mRNA purified were further analyzed using Bio-analyzer (Agilent Technologies). The protocol employed to prepare RNA-seq libraries was based on the use of Illumina TruSeq Total RNA Sample Prep kits. The cluster generation and sequencing were carried out by standard procedures in HiSeq 2000 Illumina platform with pair-end 150 bp (PE 150) sequencing strategy.

The paired-end sequences were mapped to human genome (hg38) using MapSplice 2.0.1.6 (default parameters) to discover splicing junctions. Further analyzed the mapped reads with Cufflinks can calculate the level of gene expression. We analyzed the changes of splicing isoforms using MISO package with annotation of all known AS events, and the results filtered according to the percent-spliced in values.

We performed gene ontology analysis using DAVID GO analysis software to search for enriched pathways. The functional association of Paclitaxel-induced targets were resolved using protein interaction data from STRING database, and we investigated the generating functional interaction networks. The sub-network containing more than five nodes were demonstrated.

The RNA-seq dataset was deposited to the Gene Expression Omnibus with accession number

### Cytotoxicity assay and inhibitory concentration 50 measurement

Cell growth determination kit (MTT) was used to detect the IC50. Briefly, cells were separately seeded into 96-well plates (4000 cells/well) to culture overnight and then treated with paclitaxel at different concentrations (0, 5, 10, 20, 50, 70, 100 nM) in 100 µl culture medium. After 48 h culture, 10 µl MTT solution was added and incubated for 4 h at 37 °C. Spectrophotometrically measure absorbance at a wavelength of 570 nm was performed. Dose–response curves were plotted to determine half maximal IC50 of paclitaxel using the Graphpad Prism6.

### Colony formation assay

A549 and H1299 cells or stable expression ECT2-L/S cells treated without or with stepwise concentrations of paclitaxel 24 h were seeded in 60-mm dishes (300 cells per dish) and incubated at 37 °C, 5% CO_2_ in humidified incubator for 2 weeks (updated with fresh medium every 4 days). Each treatment was carried out in triplicate. Colonies were fixed with 4% paraformaldehyde and stained with crystal violet solution. Numbers of colonies was counted.

### Immunofluorescence staining

A549 and H1299 cells stably expression pCDH-ECT2-L/S were cultured on sterile glass cover slips, treated with 7.22 nM and 40 nM paclitaxel 12 h at 37 °C. Cells were washed briefly with PBS and then fixed 10 min with methanol. Blocking with 3% BSA in PBS for 20 min after PBS washing. Rinse sections in PBS-Tween 20 for 10 min. PBS wash and then incubated with primary antibody (Flag antibody) overnight at 4 ℃. Incubated with anti-Mouse secondary antibody. Counterstain with DAPI and then captured images with a fluorescent microscope.

### Real-time cell analysis by RTCA

The RTCA instrument was used to assess the cell viability, proliferation, and migration of A549, H1299, and stably expression ECT2-L/S cells or cells treated with different concentration of Paclitaxel for 24 h. Cells were digested with trypsin briefly, counted and then re-suspended in culture medium. Background measurements were taken from the wells by adding 50 µl of the same medium to the E-16 plates. Subsequently, cells were plated at a density of 2000/well with fresh medium to a final volume of 150 µl. Cells were incubated for 30 min at room temperature and then analyzed in the RTCA in 5% CO2 and 37 °C incubator. The impedance signals were recorded every 5 min until the end of the experiment (up to 100 h). The migration assay was also tested by using cell invasion and migration plates (CIM-plates). Cell index for real-time dynamic assessment and slope calculations for the migration assessments were calculated automatically by the RTCA Software Package 1.2 of the RTCA system.

### Statistical analysis

Differences between experimental groups were evaluated by one-way ANOVA or Student test using Graphpad Prism 6 software package to analyze colony formation, cell cycle, apoptosis, and splicing changes. Statistical significance was based on a *p*-value of 0.05.

## Electronic supplementary material

Supplemental figures and table
