# Supplementary material for: Modulation of alternative splicing induced by paclitaxel in human lung cancer
Source: Cell Death Dis. 2018 Apr 30;9(5):491. doi: 10.1038/s41419-018-0539-4 (PMC5924756; doi:10.1038/s41419-018-0539-4)
Supplement: Supplementary file 1 — Supplemental figures and table [file 41419_2018_539_MOESM1_ESM.pdf]

Supplemental Figures and Table

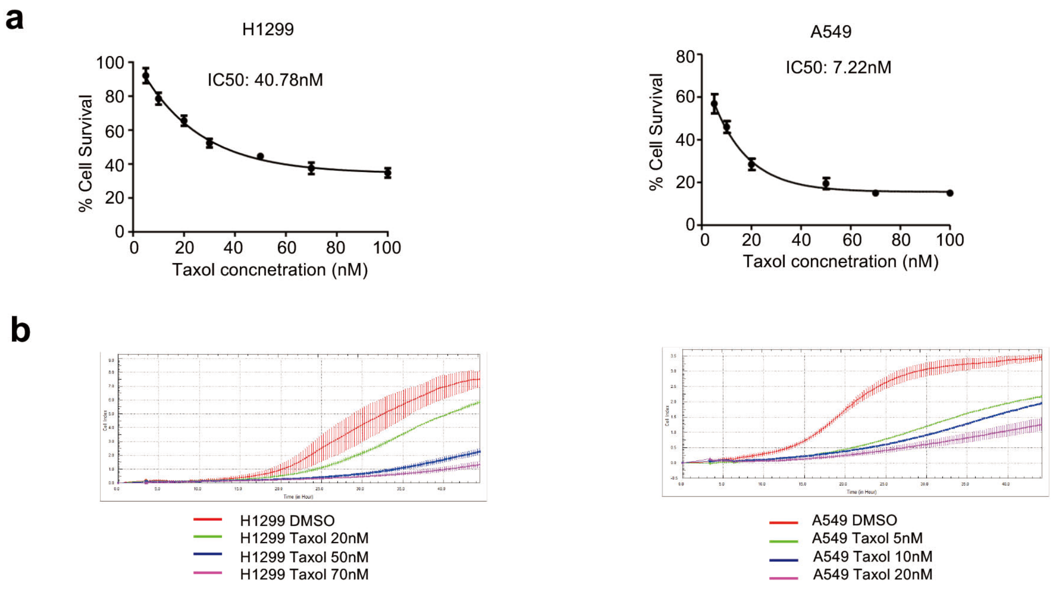

Supplemental Figure 1. The effects of paclitaxel on lung cancer cell viability and migration. (a) A549 and H1299 cells were treated with gradient concentration Paclitaxel at 0, 5, 10, 20, 50, 70, 100nM, and cell viability was tested by MTT assay 24,48h after treatment. The relative cell viability represents a ratio of Paclitaxel treatment versus control. IC50 were calculated using Graphpad Prism6. (b) Cell migration of A549 and H1299 cells was measured by RTCA assay after Paclitaxel (5, 10, 20nM) and (20, 50, 70nM) respectively for 24h.

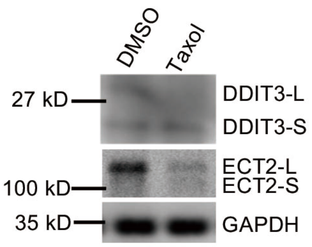

Supplemental Figure 2. The splicing changes of DDIT3 and ECT2 at protein levels. When treated with DMSO control or taxol, the splicing changes of DDIT3 and ECT2 were determined by western blot using anti-DDIT3 and anti-ECT2 antibodies.

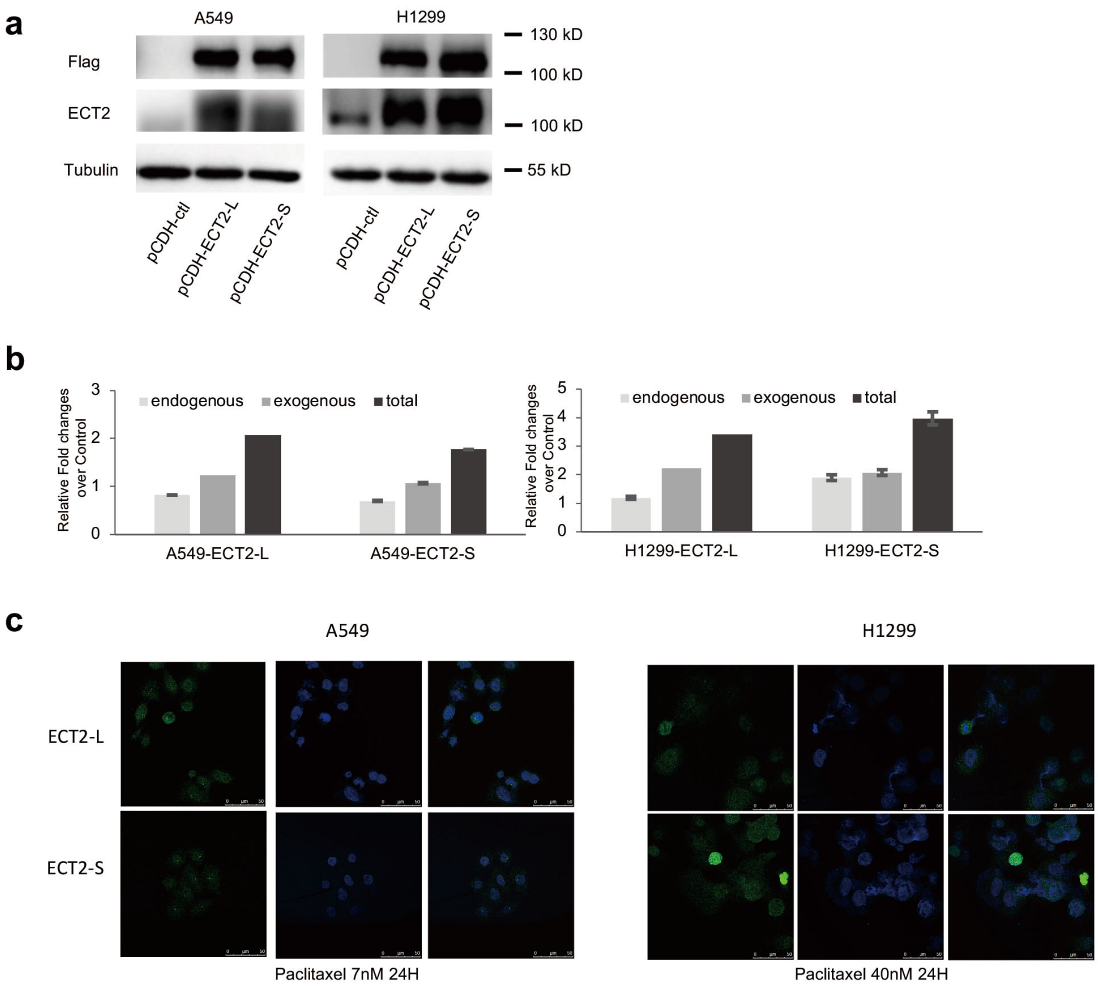

Supplemental Figure 3. The localization of ECT2-L and ECT2-S. (a) A549 and H1299 cells with stable expression of ECT2-L or ECT2-S were generated. Protein levels of exogenous and endogenous ECT2 were examined by western blot analysis using anti-Flag and anti-ECT2 antibodies. (b) Different primers were utilized for realtime PCR to show exogenous, and total expression levels of ECT2 in the cells. The endogenous level of ECT2 was calculated by subtracting exogenous from the total level of ECT2. The mean and SD of relative fold changes from triplicate experiments were plotted. (c) The localization of ECT2-L and ECT2-S was identified by Immunofluorescence with Flag antibody in Paclitaxel treated cells.

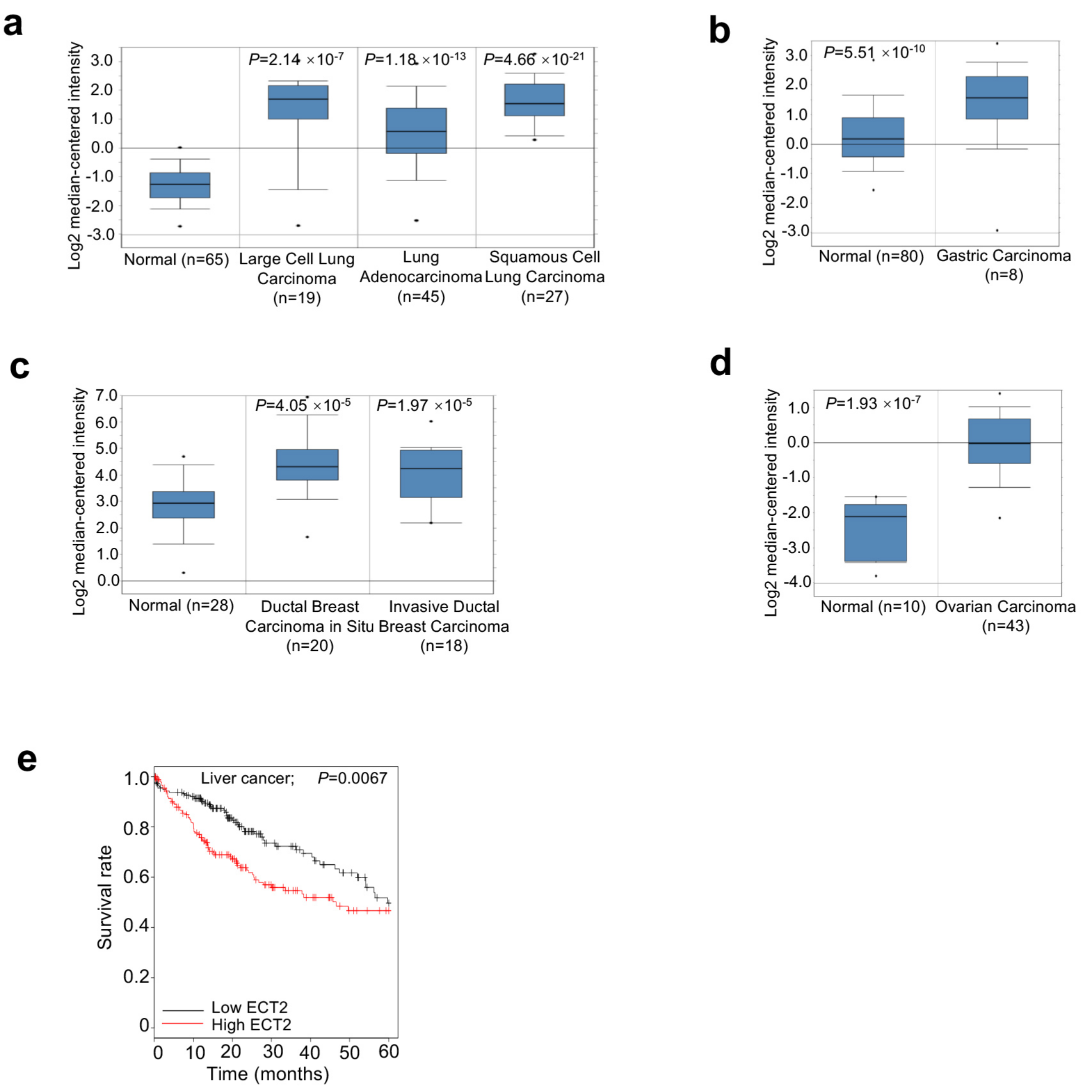

Supplemental Figure 4. The expression level and survival correlation of ECT2-L in multiple cancers. The expression levels of ECT2-L were analyzed in various large scale studies obtained from TCGA dataset, including lung (a), gastric (b), breast (c), and ovarian (d) cancers. (e) Kaplan-Meier curve showing overall survival of patients with liver cancer bearing high or low ECT2-L expression.

Supplemental Table 1: List of primers used in the study.

Primer#	Name	Sequence	Nots
1	FMNL3 AS Fwd	GTGGGTGCGGGAATTTCTGA	The primer pair used for testing SE of FMNL3
2	FMNL3 AS Rev	CGTGGACGTCATCCTTCTGG	
3	ZMIZ2 AS Fwd	CAAGGAGGCCAGTATGCACC	The primer pair used for testing SE of ZMIZ2
4	ZMIZ2 AS Rev	CAGTGGGGAACTGGGATAGC	
5	ECT2 AS Fwd	AGAGGAGTCGGCGTTTGAAG	The primer pair used for testing SE of ECT2
6	ECT2 AS Rev	AGAGTCTGCCAAGCTAGTCC	
7	PLD2 AS Fwd	GATCGAGGACACAGAGACGG	The primer pair used for testing 5'ss usage of PLD2
8	PLD2 AS Rev	CAGCCATGTCTTGCCACAAC	
9	ELF2 AS Fwd	TCTCCTACCTGCTTGAGGGA	The primer pair used for testing 3'ss usage of ELF2
10	ELF2AS Rev	ACTCTTCAGTTGACACCTCCAC	
11	DDIT3 AS fwd	CATACATCACCACACCTGAAAGC	The primer pair used for testing 3'ss usage of DDIT3
12	DDIT3 AS Rev	AGTGTCCCGAAGGAGAAAGG	
13	PLK1 Fwd	TGACTCAACACGCCTCATCC	The primer pair used for testing PLK1
14	PLK1 Rev	GCTCGCTCATGTAATTGCGG	
15	SQSTM1 Fwd	TAGGAACCCGCTACAAGTGC	The primer pair used for testing SQSTM1
16	SQSTM1 Rev	CTGGTGGACCCATTTCCCAT	
17	ATF3 Fwd	TTTTGCTAACCTGACGCCCT	The primer pair used for testing ATF3
18	ATF3 Rev	TCTTGTTTCGGCACTTTGCAG	
19	IL1B Fwd	GGGCCTCAAGGAAAAGAATC	The primer pair used for testing IL1B
20	IL1B Rev	TTCTGCTTGAGAGGTGCTGA	
21	SAT1 Fwd	GCAGTGACATACTGCGGCTG	The primer pair used for testing SSAT1
22	SAT1 Rev	CGGAGTCCAGTGCTCTTTCG	
23	SOD2 Fwd	GGCCTACGTGAACAACCTGA	The primer pair used for testing SOD2
24	SOD2 Rev	GTTCTCCACCACCGTTAGGG	
25	AURKA Fwd	CTTCCCAGCGCATTCCTTTG	The primer pair used for testing AURKA
26	AURKA Rev	TTCCTTTACCCAGAGGGCGA	
27	CDCA8 Fwd	ACATCTCAGGGAATGGCAGC	The primer pair used for testing CDCA8
28	CDCA8 Rev	TGGGCAATACTGTGCCTCTG	
29	GADD45A Fwd	GTCGGGGTGTACGAAGCG	The primer pair used for testing GADD45A
30	GADD45A Rev	TTCTCGCAGCAAAACGCCT	
31	CDKN1A Fwd	TCTTACAAACCGAACTTGGC	The primer pair used for testing CDKN1A
32	CDKN1ARev	GGCGGTACTGTGAAGACGTT	
33	FOXM1 Fwd	TGGGGAGGAAATGCCACACTTA	The primer pair used for testing FOXM1
34	FOXM1 Rev	TAGGACTTCTTGGGTCTTGGGG	
35	ECT2 3*FLAG CDS Fwd	CCGGGATCCGCTGAAAATAGTG TATTAACATCC	The primer pair used for cloning ECT2 coding region into pCDH vector with 3* FLAG epitope
36	ECT2 CDS Rev	CCGGCGGCCGCTCATATCAAA TGAGTTGTAG	
37	HNRNPUL1 Fwd	AGATGAAGCAAGGAGCACCC	The primer pair used for testing hnRNPUL1
38	HNRNPUL1 Rev	CTTGAAGTGGAGGTCGCAGT	
